# Different degrees of nodes behind obsessive–compulsive symptoms of schizophrenia

**DOI:** 10.3389/fpsyt.2023.1224040

**Published:** 2023-07-27

**Authors:** Yiying Hu, Xiaopei Xu, Liyuan Luo, Huichao Li, Wangtao Li, Liyuan Guo, Lanying Liu

**Affiliations:** ^1^Department of Psychiatry, Shanghai Mental Health Center, Shanghai Jiao Tong University School of Medicine, Shanghai, China; ^2^Zhejiang Chinese Medical University, Hangzhou, China; ^3^Department of Radiology, The Second Affiliated Hospital of Zhejiang University School of Medicine, Hangzhou, China; ^4^Tongde Hospital of Zhejiang Province, Hangzhou, China; ^5^Shanghai Institute of Mental Diseases of Traditional Chinese Medicine, Shanghai, China

**Keywords:** obsessive–compulsive symptoms of schizophrenia, brain network, network analysis, diffusion tensor imaging, degree of a node

## Abstract

Obsessive–compulsive symptoms are frequently observed in various psychiatric disorders, including obsessive–compulsive disorder, schizophrenia, depression, and anxiety. However, the underlying anatomical basis of these symptoms remains unclear. In this study, we aimed to investigate the mechanism of schizophrenia with obsessive–compulsive symptoms by using diffusion tensor imaging (DTI)-based structural brain connectivity analysis to assess the network differences between patients with obsessive–compulsive disorder (OCD), patients with schizophrenia showing obsessive–compulsive symptoms (SCH), schizophrenia patients with obsessive–compulsive symptoms due to clozapine (LDP), and healthy controls (CN). We included 21 patients with OCD, 20 patients with SCH, 12 patients with LDP, and 25 CN. All subjects underwent MRI scanning, and structural brain connections were estimated using diffusion tensor imaging for further analysis of brain connectivity. The topology and efficiency of the network and the characteristics of various brain regions were investigated. We assessed baseline YALE-BROWN OBSESSIVE COMPULSIVE SCALE (Y-BOCS), Positive and Negative Syndrome Scale (PANSS), and 24-item Hamilton Depression Scale (HAMD-24) scores. Our results showed significant differences among the SCH, OCD, and CN groups (*p* < 0.05) in the MRI-measured degree of the following nodes: the superior orbitofrontal gyrus (25Frontal_Med_Orb_L), lingual gyrus (47Lingual_L), postcentral gyrus (58Postcentral_R), and inferior temporal gyrus (90Temporal_Inf_R). Additionally, we found significant differences in the degree of the brain regions 02Precentral_R, 47Lingual_L, 58Postcentral_R, and 90Temporal_Inf_R between the CN, OCD, SCH, and LDP groups (*p* < 0.05). These findings suggest that alterations in the degree of nodes might be the mechanism behind obsessive–compulsive symptoms in schizophrenia.

## Introduction

Obsessions are characterized by recurring opinions, thoughts, impulses, or mental images, while compulsive behavior involves purposeful and conscious actions that are repeated; collectively, obsessions and compulsions are known as obsessive–compulsive symptoms. These symptoms are prevalent not only in individuals with obsessive–compulsive disorder but also in those with other conditions such as depression, anxiety, and schizophrenia. Obsessive–compulsive symptoms in schizophrenia have long been noted, but the current understanding is still very limited. According to recent reports, the prevalence of obsessive–compulsive symptoms in patients with schizophrenia is estimated to be 30% (1), and the risk of obsessive–compulsive disorder is more than 10 times higher in these patients than in the general population (2). However, the results of studies on this topic have been highly variable, with several studies appearing to contradict the finding of 30%. For instance, Baek et al. (3) conducted a survey of 320 clinically stable patients with chronic schizophrenia and found that the incidence of obsessive–compulsive disorder was 20.6%. Similarly, Singh et al. (4) reported a prevalence of obsessive–compulsive symptoms of 18.5% among 200 outpatients with schizophrenia. In another cross-sectional study conducted by Ahmet et al., which included 300 patients with schizophrenia, the overall prevalence of obsessive–compulsive disorder (OCD) was 17% (5).

The lack of a standardized assessment of obsessive–compulsive symptoms in schizophrenia may have contributed to the variability in the reported prevalence rates among different studies. Moreover, the underlying mechanism of these symptoms in schizophrenia remains unclear. While genetic factors are known to play a crucial role in both schizophrenia and obsessive–compulsive disorder, there is insufficient evidence to support the notion that any specific genetic variant increases the risk of either disorder.

The catechol O-methyltransferase (COMT) gene has been implicated in both schizophrenia and obsessive–compulsive disorder. Pooley et al. (6) also showed through a case–control study and meta-analysis that the association between the Met allele and OCD existed only in men. The COMT Val105/158Met polymorphism has been suggested to be a cause of schizophrenia due to its effect on dopamine metabolism, as supported by several studies (7). Recent research has shown that SCZ patients exhibit high COMT activity and low dopamine levels in the prefrontal cortex. This leads to persistently unstable and inefficient activity of the prefrontal cortex network, which may be a mechanism underlying the involvement of COMT in schizophrenia (8). Nonetheless, the lack of a standardized assessment of obsessive–compulsive symptoms in schizophrenia may have contributed to variations in study results. Although genetic factors are thought to play a crucial role in both schizophrenia and obsessive–compulsive disorder, there is currently insufficient evidence to suggest that any particular genetic variant increases the risk of either disorder.

Regarding neuroimaging, studies using gray matter structural MRI have shown that patients with schizophrenia and obsessive–compulsive disorder exhibit abnormal gray matter structure, but the specific results of these studies have been inconsistent. Kwon et al. (9) reported that patients with both disorders had smaller bilateral hippocampi than normal controls, suggesting that this may be related to overlapping symptoms between the two conditions. Meanwhile, Opel et al. (10) conducted a comparison of brain structure among patients with six common mental disorders and found a strong correlation (*r* = 0.443–0.782) in brain structure abnormalities among patients with schizophrenia, obsessive–compulsive disorder, major depression, and bipolar disorder. They also noted that patients with schizophrenia and obsessive–compulsive disorder showed abnormalities in morphological characteristics of brain regions such as the hippocampus and fusiform gyrus.

Several studies using diffusion tensor imaging (DTI) have shown that patients with various psychiatric disorders, including schizophrenia and obsessive–compulsive disorder, frequently exhibit abnormalities in the white matter structure of the brain, particularly in the cingulate bundle, corpus callosum, and white matter fiber bundles in the frontal and temporal regions (11–13). Hawco et al. (14) found that the FA value of the corpus callosum decreased in patients with schizophrenia and obsessive–compulsive disorder compared to the normal control group, while the difference in total white matter FA value between the two groups was not statistically significant, suggesting that schizophrenia and obsessive–compulsive disorder may have a common neurobiological substrate. Wang et al. (15) found abnormalities in the FA value and radial diffusion (RD) of brain white matter in patients with obsessive–compulsive disorder and schizophrenia, while no significant changes were found in these variables in patients with schizophrenia or obsessive–compulsive disorder. Cauda et al. found that the brains of patients with both schizophrenia and obsessive–compulsive disorder exhibit common atrophy of structural networks (reduced gray matter volume) in brain connectome analyses. However, previous studies comparing the brain connectomes of people with schizophrenia and OCD have been limited and used different methods, thus drawing no firm conclusions (16).

The structural brain network is a complex system of connections between different regions of the brain that are responsible for various functions. These connections can be studied using techniques such as DTI, which measures the diffusion of water molecules in the brain’s white matter tracts. The structural brain network is constructed by analyzing the patterns of connectivity between different regions of the brain. This analysis involves identifying the nodes (regions of the brain) and edges (connections between nodes) that make up the network. The topology of the network can then be analyzed to understand the efficiency and resilience of the network (17). The clinical significance of analyzing the structural brain network lies in its potential to provide insights into the underlying mechanisms of various neurological and psychiatric disorders. For example, studies have shown that alterations in the structural brain network are associated with conditions such as Alzheimer’s disease, schizophrenia, and depression (18, 19). By understanding the changes in the structural brain network associated with these disorders, researchers may be able to develop more effective treatments and interventions. Additionally, analyzing the structural brain network may help identify biomarkers that can be used to diagnose and monitor the progression of these disorders (20).

Antipsychotics are primarily blockers of DA and 5-HT receptors, which may lead to the development of obsessive–compulsive symptoms due to long-term 5-HT/DA dysfunction (21). It has been observed that obsessive symptoms may emerge after prolonged use of antipsychotics, particularly clozapine. The incidence of obsessive–compulsive symptoms induced or aggravated by clozapine is reported to be between 10 and 20.6% among patients with schizophrenia using clozapine (22). Clozapine has been found to induce obsessive–compulsive symptoms in patients with schizophrenia, which could be due to its potent blockade of the 5-HT2A receptor. Nevertheless, the relationship between dose and symptoms remains controversial, and the mechanism underlying the emergence of these symptoms requires further investigation.

Studying the mechanism behind the formation of obsessive–compulsive symptoms in schizophrenia and in patients taking clozapine is crucial to distinguish them from obsessive–compulsive disorder and to reduce misdiagnosis rates. The advancement of brain imaging technology, particularly magnetic resonance imaging (MRI), has provided an essential tool to gain further insight into schizophrenia and obsessive–compulsive disorder. One of the most promising and innovative research approaches in the field of brain science is brain connectome analysis, which examines both the functional and structural networks of the brain, primarily driven by the models and methods of graph theory. Consequently, the present study utilized this method for analysis.

## Materials and methods

### Participants

The participants were randomly selected outpatients and inpatients from Tongde Hospital in Zhejiang Province, Hangzhou, China. The subjects signed informed consent forms. All participants had a YBOCS score ≥ 16, all were between the ages of 16 and 60 years, and all were right-handed. Participants with obsessive–compulsive disorder (OCD) met the Diagnostic and Statistical Manual of Mental Disorders-Fifth Edition, DSM-V criteria for obsessive–compulsive disorder. Participants with schizophrenia showing obsessive–compulsive symptoms (SCH) met the DSM-V diagnostic criteria for schizophrenia, and their obsessive–compulsive symptoms had appeared before antipsychotic treatment. Schizophrenia participants with obsessive–compulsive symptoms caused by clozapine (LDP) met the DSM-V diagnostic criteria for schizophrenia, had no obsessive–compulsive symptoms before treatment, had obsessive–compulsive symptoms for at least 2 weeks after starting treatment with clozapine, and had stable mental symptoms. The exclusion criteria were as follows: ① Compulsive symptoms secondary to organic brain diseases, especially basal ganglia diseases; ② Obsessive–compulsive disorder in conjunction with severe cardiac, hepatic, or renal insufficiency; ③ Received modified electric convulsive therapy within the past 6 months; ④ Alcohol or drug dependence; ⑤ Pregnancy at the time of the study; and ⑥ Left-handedness. CN patients matched by age, gender, and education level were recruited from the community, and informed consent was obtained. The exclusion criteria were the same for the control group as for the OCD, SCH, and LDP groups. The study was approved by the Ethics Committee of Tongde Hospital, Zhejiang Province. All participants provided written informed consent prior to study entry. Ultimately, a total of 21 patients for the OCD group (30.38 ± 13.56), 20 patients for the SCH group (34.75 ± 6.36), 12 patients for the LDP group (30.50 ± 6.54), and 25 healthy controls (CN; 31.48 ± 5.34) were included.

### Image acquisition

All MR images were acquired at the Department of Radiology of Tongde Hospital utilizing a 3.0-Tesla scanner (Magnetom Trio Tim, Siemens). To minimize head movement, a 16-channel birdcage head coil with foam pads was used to secure each subject’s head. A gradient echo sequence was employed to acquire 3D T1-weighted images with isotropic 1 mm^3^ voxels; the scan covered the entire brain and proceeded parallel to the anterior commissure–posterior commissure (AC–PC) line. The following parameters were used: flip angle = 9°; TR = 1900 ms, TE = 2.48 ms, TI = 900 ms; slice thickness = 1.0 mm without gaps; reconstruction resolution = 1 ×  1 × 1 mm^3^; field of view = 512 × 512 mm^2^. The diffusion-weighted imaging (DWI) data were composed of a non-diffusion-weighted image (b0) and diffusion-weighted images along 30 gradient directions with a *b*-value of 1,000 s/mm^2^. A single-shot echo-planar sequence was used to acquire DWI data with the following parameters: TR/TE = 8,600/92 ms; number of transversal slices = 55; flip angle = 90°; NEX = 1; gradient directions = 30; field of view = 256 × 248 mm^2^; and slice thickness = 2.0 mm without gaps.

### Image processing and brain connectivity analysis

The following section outlines the comprehensive procedures for image processing and brain connectivity analysis.

### Brain network construction

#### Brain parcellation

Anatomical referencing and parcellation of the brain into 90 cortical and subcortical regions were performed for each subject using T1W images and the automated anatomical labeling (AAL) atlas. The following steps were taken: (1) FLIRT was used to register T1W images to DWI in the native space. (2) FNIRT was employed to map the registered structural images to the ICBM 152 template. (3) The estimated transformation parameters were inverted and applied to the AAL atlas to warp all brain regions of interest from MNI space to the native diffusion space.

#### White matter tractography

To correct for head motions and eddy currents, FMRIB’s Diffusion Toolbox was utilized to register all DWI scans to b0. The linear least-squares fitting method was employed to estimate the diffusion tensor for each voxel using Diffusion Toolkit. Subsequently, tractography was performed using the fiber tracking algorithm with a fractional anisotropy threshold of 0.2 and a fiber turning angle threshold of 45° using Diffusion Toolkit to construct the structural connections between the 90 brain regions.

#### Brain network

In a brain network, each brain region is regarded as a node, and each connection between regions is considered an edge. If WM fiber tracts originate from one region and terminate in another, the connections between the 90 brain regions are considered edges. Using the UCLA Multimodal Connectivity Package in the native diffusion space (23), we estimated the number of fibers between regions and weighted each edge accordingly. An inter-regional undirected network with weighted connections was thus obtained for each subject.

### Brain network analysis

To minimize the overall differences in connectivity strength, each weighted connectivity matrix was normalized to its maximum fiber count. The Brain Connectivity Toolbox was utilized to estimate the properties of each participant’s global network, such as the small-world properties and network efficiency, as well as the nodal characteristics of each brain region in each subject’s normalized connectivity matrix.

#### Small-world properties

The clustering coefficient measures the likelihood that a node’s neighborhoods are connected to each other, and the clustering coefficient of the entire network reflects the extent of the local cluster in a network. Characteristic shortest path length measures the shortest geodesic length between a node and any other node, and that of the entire network is the average shortest path length between all pairs of nodes. To determine whether a network is a small-world network, the clustering coefficient and characteristic shortest path length of the network are compared with those of random networks (11). We generated 100 randomized networks with the same number of nodes, number of edges, and degree distribution as the original network. A network is considered a small-world network when the normalized clustering coefficient ( γ ) is much greater than one and the normalized characteristic shortest path length ( λ ) is close to one.

#### Network efficiencies

The global efficiency is calculated as the average of the inverse of the shortest path length for all node pairs in the entire network (24). The local efficiency of the entire network can be determined by averaging the global efficiency values of all subnetworks. It measures the fault tolerance of the entire network and reflects how well each subnetwork exchanges information when the most connected node is removed.

#### Nodal characteristics

A node’s degree is the number of edges that connect it to the rest of the network. The nodal efficiency of a given node is defined as the inverse of the harmonic mean of the characteristic shortest path length between it and all other nodes in the entire network. The betweenness centrality of a given node quantifies the number of shortest paths between nodes in the entire network that pass through the given node. It is a measure of the node’s importance for the integration of all the connections among all nodes in the network.

### Statistical analysis

Comparisons between the network measures of all cohorts were performed using one-way ANOVA followed by Bonferroni *post hoc* analysis. Pearson correlations were also performed to determine the association between network measures and clinical assessments. All analyses were corrected for age and sex. A significance threshold of *p* < 0.05 was set for all statistical tests. All statistical analyses were performed using SPSS 22.0 (SPSS, Chicago, IL, United States).

## Results

### Demographic and clinical characteristics

The demographics and clinical characteristics of the participants are shown in Table 1. There were no significant differences in age, gender, or, where applicable, total illness duration among CN and patients with OCD, SCH, and LDP.

**Table 1:** Demographic and clinical characteristics of CN and OCD, SCH and LDP patients.

	OCD	SCH	LDP	CN	*t/χ^2^*	*p*
Sample size	21	20	12	25	-	-
Age (years, mean ± SD)	30.38 ± 13.56	34.75 ± 6.36	30.50 ± 6.54	31.48 ± 5.34	1.05	0.38
Gender (male/female)	8/13	6/14	3/9	8/17	0.21	0.89
Marital status (single/married/divorced/widowed)	11/10/0/0	4/16	8/4/0/0	10/15	2.79	0.05
Total illness duration (months)	35.95 ± 52.74	60.15 ± 53.17	46.42 ± 73.36	-	0.89	0.42
Age of onset (years)	26.24 ± 11.76	30.05 ± 5.52	26.75 ± 5.45	-	1.13	0.33
Obsessive thinking total	11.33 ± 4.26	14.35 ± 2.70	15.50 ± 2.97	-	6.71	0.00
Obsessive action total	8.81 ± 5.11	6.60 ± 3.76	5.17 ± 3.41	-	3.02	0.06
Y-BOCS total	20.62 ± 5.01	21.00 ± 2.97	20.67 ± 1.87	-	0.06	0.94
PANSS total	31.86 ± 10.62	68.90 ± 14.03	57.83 ± 13.72	-	45.38	0.00
HAMD total	21.00 ± 10.34	11.15 ± 7.20	9.42 ± 4.40		10.64	0.00

OCD, obsessive–compulsive disorder; SCH, schizophrenia; LDP, schizophrenia with obsessive–compulsive symptoms caused by clozapine; CN, healthy controls; −, not applicable.

### Analysis of the MR-derived degree of nodes among different groups

**Figure 1:**
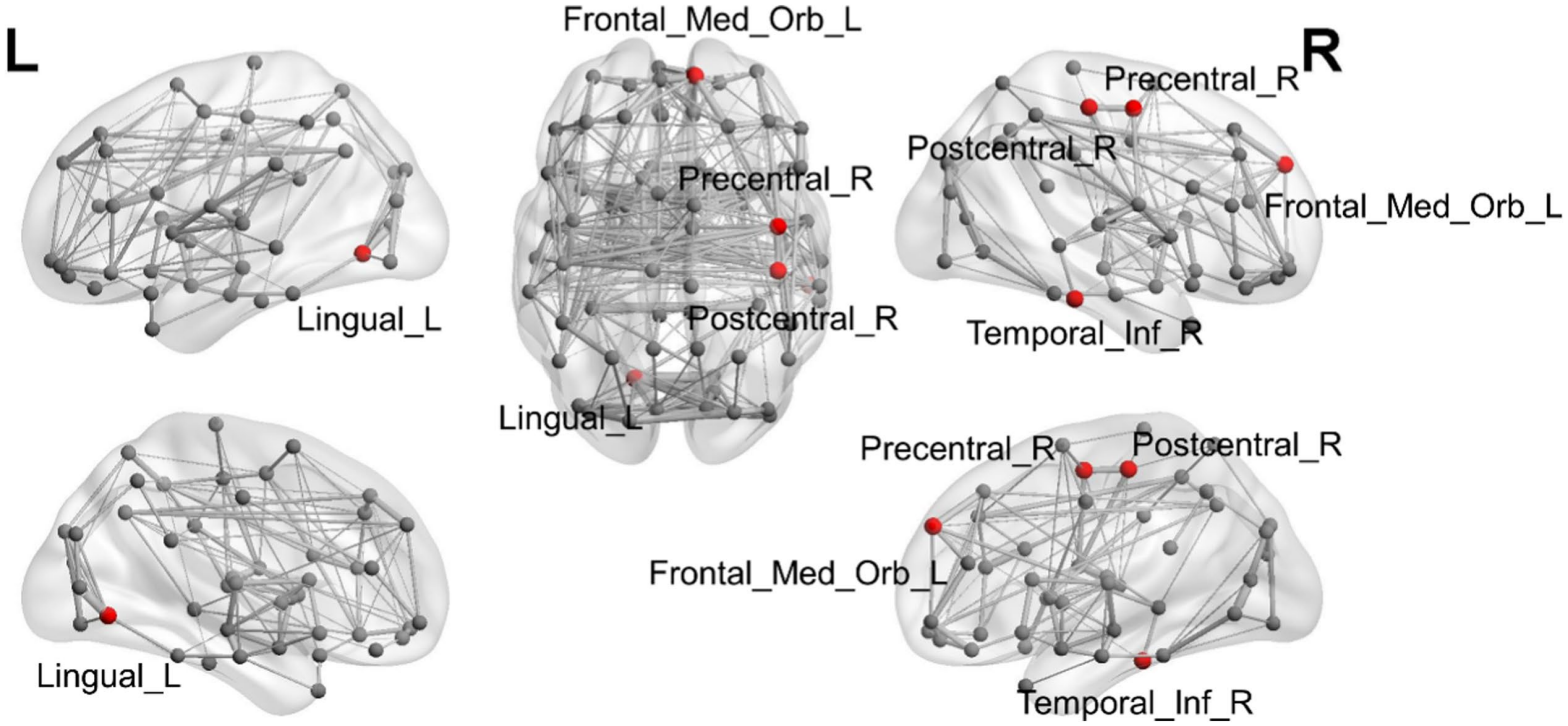
Illustration of the brain regions with significant differences in degree among CN and OCD, SCH, and LDP patients.

ANOVA was used to study the differences between groups in the precentral gyrus (02Precentral_R), superior orbitofrontal gyrus (25Frontal_Med_Orb_L), lingual gyrus (47Lingual_L), postcentral gyrus (58Postcentral_R), and inferior temporal gyrus (90Temporal_Inf_R; Table 2; Figure 1). The MR-derived degrees of the brain regions 02Precentral_R, 25Frontal_Med_Orb_L, 47Lingual_L, 58Postcentral_R, and 90Temporal_Inf_R were significantly different among the CN, OCD, SCH, and LDP groups (*p* < 0.05).

**Table 2:** ANOVA of MR degree among CN and OCD, SCH, and LDP patients.

Brain region	Group	Sample size	Mean deviation	Standard deviation	*F*	*p*
02Precentral_R	LDP	12	15.75	2.86	2.855	0.043^*^	CN	25	18	2.8	OCD	21	18.52	3.12	SCH	20	17.55	1.88	Total	78	17.68	2.8
25Frontal_Med_Orb_L	LDP	12	10	3.81			3.109	0.031^*^	CN	25	11.2	2.61	OCD	21	8.52	3.27	SCH	20	9.35	2.89	Total	78	9.82	3.19
47Lingual_L	LDP	12	18.75	6.21					3.782	0.014^*^	CN	25	16.88	3.83	OCD	21	14.24	2.91	SCH	20	16.85	3.15	Total	78	16.45	4.12
58Postcentral_R	LDP	12	17.5	3.71							3.06	0.033^*^	CN	25	19.16	3	OCD	21	20.71	3.99	SCH	20	18.2	2.67	Total	78	19.08	3.46
90Temporal_Inf_R	LDP	12	13.25	2.93									3.047	0.034^*^	CN	25	14.92	4.54	OCD	21	17.24	4.68	SCH	20	14.1	3.64	Total	78	15.08	4.32

* Significantly correlated at the 0.05 level (two-tailed test).

Brain regions with significantly different degrees are indicated in red. The node size and edge width are weighted by the nodal efficiency and number of connections, respectively.

**Figure 2:**
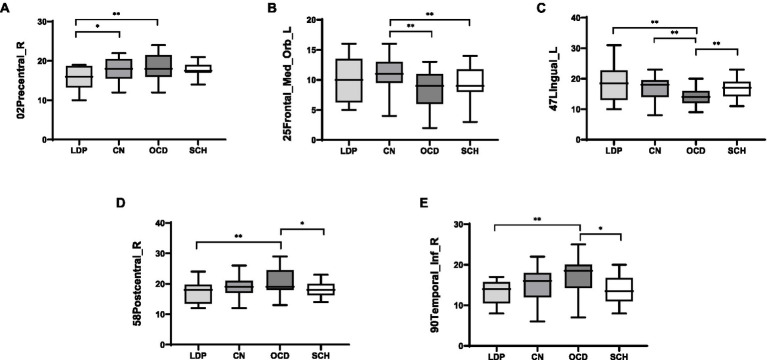
Boxplots (mean ± SD) of the MR-measured degree in the brain regions 02Precentral_R, 25Frontal_Med_Orb_L, 47Lingual_L, 58Postcentral_R, and 90Temporal_Inf_R in LDP, OCD, and SCH patients and CN. **(A)** For 02Precentral_R, compared with LDP patients, significantly increased MR-measured degree was observed in OCD patients (*p* = 0.006) and CN (*p* = 0.02). **(B)** For 25Frontal_Med_Orb_L, compared with CN, significantly reduced degree was observed in patients with OCD (*p* = 0.004) and SCH (p = 0.004). **(C)** For 47Lingual_L, compared with OCD patients, significantly increased degree was observed in patients with LDP (*p* = 0.002), SCH (*p* = 0.0036), and CN (*p* = 0.0026). **(D)** For 58Postcentral_R, compared with OCD patients, significantly reduced degree was observed in patients with LDP (*p* = 0.009) and SCH (*p* = 0.018). **(E)** For 90Temporal_Inf_R, when compared with OCD patients, significantly reduced degree were observed in patients with LDP (*p* = 0.01) and SCH (*p* = 0.018). ^*^Significantly correlated at the 0.05 level (two-tailed test). ^**^Significantly correlated at the 0.01 level (two-tailed test).

For 02Precentral_R, SCH patients showed no significant difference in MR-measured degree from the other groups; compared with OCD patients, significantly reduced degree was observed in patients with LDP (*p* = 0.006), and significantly increased degree was observed in CN (*p* = 0.02; Table 3; Figure 2). For 25Frontal_Med_Orb_L, compared with SCH patients, a significantly increased degree was observed in patients with CN (*p* = 0.004), and no significant difference in MR degree was observed between the remaining two groups; compared with OCD patients, a significantly increased degree was observed in CN (*p* = 0.004). For 47Lingual_L, compared with SCH patients, a significantly reduced degree was observed in patients with OCD (*p* = 0.0036), and no significant difference in degree was observed in the remaining two groups; compared with OCD patients, a significantly increased degree was observed in LDP patients (*p* = 0.002) and CN (*p* = 0.0026). For 58Postcentral_R, compared with SCH patients, a significantly increased MR degree was observed in patients with OCD (*p* = 0.018), and no significant difference in MR degree was observed in the remaining two groups; compared with OCD patients, a significantly reduced degree was observed in patients with LDP (*p* = 0.009). For 90Temporal_Inf_R, compared with SCH patients, a significantly increased degree was observed in patients with OCD (*p* = 0.018), and no significant difference in degree was observed in the remaining two groups; compared with OCD patients, a significantly reduced MR degree was observed in patients with LDP (*p* = 0.01).

**Table 3:** *Post hoc* analysis of MR-measured degree among OCD, SCH, and LDP patients and CN.

Brain region	Group 1	Group 2	Mean deviation 1	Mean deviation 2	Difference	*p*
02Precentral_R	LDP	CN	15.75	18	−2.25	0.02^*^
	LDP	OCD	15.75	18.524	−2.774	0.006^*^
	LDP	SCH	15.75	17.55	−1.8	0.072
	CN	OCD	18	18.524	−0.524	0.515
	CN	SCH	18	17.55	0.45	0.581
	OCD	SCH	18.524	17.55	0.974	0.252
25Frontal_Med_Orb_L	LDP	CN	10	11.2	−1.2	0.269
	LDP	OCD	10	8.524	1.476	0.187
	LDP	SCH	10	9.35	0.65	0.563
	CN	OCD	11.2	8.524	2.676	0.004^*^
	CN	SCH	11.2	9.35	1.85	0.048
	OCD	SCH	8.524	9.35	−0.826	0.391
47Lingual_L	LDP	CN	18.75	16.88	1.87	0.178
	LDP	OCD	18.75	14.238	4.512	0.002^*^
	LDP	SCH	18.75	16.85	1.9	0.188
	CN	OCD	16.88	14.238	2.642	0.026^*^
	CN	SCH	16.88	16.85	0.03	0.98
	OCD	SCH	14.238	16.85	−2.612	0.036^*^
58Postcentral_R	LDP	CN	17.5	19.16	−1.66	0.16
	LDP	OCD	17.5	20.714	−3.214	0.009^*^
	LDP	SCH	17.5	18.2	−0.7	0.566
	CN	OCD	19.16	20.714	−1.554	0.119
	CN	SCH	19.16	18.2	0.96	0.339
	OCD	SCH	20.714	18.2	2.514	0.018^*^
90Temporal_Inf_R	LDP	CN	13.25	14.92	−1.67	0.256
	LDP	OCD	13.25	17.238	−3.988	0.01^*^
	LDP	SCH	13.25	14.1	−0.85	0.577
	CN	OCD	14.92	17.238	−2.318	0.063
	CN	SCH	14.92	14.1	0.82	0.513
	OCD	SCH	17.238	14.1	3.138	0.018^*^

* Significantly correlated at the 0.05 level (two-tailed test).

## Discussion

The etiology and pathogenesis of schizophrenia remain unclear, and clinical diagnosis primarily relies on clinical manifestations, given the dearth of objective laboratory indicators. Obsessive–compulsive symptoms not only occur in patients with obsessive–compulsive disorder but also have a high incidence in several other disorders, such as depression, anxiety, and schizophrenia. Studies have reported that the prevalence of obsessive–compulsive symptoms is higher in schizophrenia patients than in the general population, and schizophrenia patients with obsessive–compulsive symptoms (OCS) tend to experience more severe symptoms and poorer prognosis than those without OCS (1). In recent years, diffusion tensor imaging (DTI) has been increasingly used to detect abnormal brain functional connectivity in patients with mental disorders, including schizophrenia and obsessive–compulsive disorder. In this study, we conducted a comparison of DTI-based functional brain imaging in patients with schizophrenia and obsessive–compulsive symptoms (SCH), clozapine-induced obsessive–compulsive symptoms (LDP), and obsessive–compulsive disorder (OCD), along with healthy volunteers (CN). Our findings demonstrated that there were significant differences in the MR degree of 25Frontal_Med_Orb_L, 47Lingual_L, 58Postcentral_R, and 90Temporal_Inf_R among the three groups (*p* < 0.05). This may have significance as a reference for neuroimaging studies of schizophrenia with obsessive–compulsive symptoms.

There is a high comorbidity rate between schizophrenia and obsessive–compulsive disorder (OCD), but the shared neurobiological substrate and the aetiological relationship between these two disorders are still unclear (25). Using DTI technology, Hawco et al. (14) found that compared with the normal control group, patients with schizophrenia and OCD had reduced FA values in the corpus callosum, and there was no significant difference in the FA values of the total white matter between the two groups, suggesting that schizophrenia and OCD may have a common neurobiological substrate. At present, there are still few studies directly comparing the brain functional magnetic resonance imaging characteristics of SZ and OCD. The majority of scholars believe that the onset of obsessive–compulsive disorder (OCD) results from an imbalance of neurotransmitter and neuronal metabolite concentrations in the cortico-striato-thalamo-cortical (CSTC) circuit, and neuroimaging studies have revealed that the pathophysiological mechanism of OCD may involve a broader range of brain regions, such as the parietal cortex and the posterior temporo-parieto-occipital junction area (26). Schizophrenia is associated with changes in the amplitude of low frequency fluctuation (ALFF) in a wide range of brain regions, such as frontal, temporal, parietal, occipital, and limbic lobes (23). Several studies have shown that patients with schizophrenia and obsessive–compulsive disorder often have abnormalities in brain white matter structure, including the cingulate tract, the corpus callosum, and white matter fiber tracts in frontal and temporal regions (11, 12).

In the brain region of 25Frontal_Med_Orb_L, which is the superior orbitofrontal gyrus, the MR-measured degree was significantly decreased in patients with SCH compared to CN (*p* = 0.004). The superior orbitofrontal gyrus is an important part of the prefrontal lobe, playing an important role in advanced cognition and autonomous behavior. This gyrus is an important part of the cognitive control network, participating in emotion regulation, execution of cognitive behaviors, memory, decision-making, and other processes (24). There is a correlation between the prefrontal lobe and obsessive–compulsive disorder. Human neuroimaging studies have consistently highlighted abnormal activity patterns in prefrontal cortex (PFC) regions and connected circuits during symptom elicitation and performance of neurocognitive tasks in OCD (27). Qin et al. (28) applied graph theory and network-based statistics (NBS) methods to DTI studies and found that the node efficiency and strength of the orbitofrontal gyrus were reduced in OCD patients. Park et al. (29) found a significant increase in white matter volume in the right dorsolateral prefrontal cortex in OCD patients compared with healthy controls, but no brain regions with reduced white matter volume were found in OCD, and no changes in gray matter volume were found between the two groups. Remijnse et al. (30) found that the orbitofrontal cortex (OFC) and striatal function were impaired in OCD. At present, there are few studies on the relationship between the superior orbitofrontal gyrus/prefrontal lobe and schizophrenia with obsessive–compulsive symptoms (OCS), and the relationship between the orbitofrontal cortex and schizophrenia with OCS has not been clarified. Schizophrenia and OCD patients commonly present abnormalities in brain white matter structure, and patients with a variety of psychiatric disorders, including schizophrenia and OCD, have an elevated risk of abnormalities in white matter fiber tracts in frontal regions (11). Zhu et al. (31) found that decreased orbital frontal cortex connectivity in schizophrenia may be related to reduced activity in the orbitofrontal cortex due to low activation of dopaminergic inputs in the endodermis. A study by Schirmbeck et al. (32) in patients with schizophrenia showed a significant increase in OFC activity during the onset of obsessive symptoms with antipsychotic medication. Increased orbitofrontal cortex activation is associated with “pro-obsessive” antipsychotic treatment in patients with schizophrenia. This is not consistent with our results. This may be related to the sample size and different demographic characteristics; further exploration and study are needed.

The brain region 47Lingual_L, which is located in the occipital lobe, had a significantly higher MR-measured degree in SCH than in OCD (*p* = 0.0036). Some scholars have found that in OCD patients, the intrinsic functions from the cortico-striato-thalamo-cortical (CSTC) circuits to the occipital lobe are changed (33). The CSTC circuit involves multiple brain regions, such as the prefrontal lobe, striatum, and thalamus, and has been shown to be closely related to the pathophysiology of OCD (34). The lingual gyrus, which is central to the visual cortex, is located in the occipital lobe. The occipital lobe is not a component of the CSTC neural circuit, and there is limited research on the occipital lobe in obsessive–compulsive disorder (35). However, the occipital lobe is not commonly associated with schizophrenia. Tohid et al. (36) found that most patients with schizophrenia exhibit normal occipital lobe anatomy and physiology. Michel et al. (37) found that the activity of xanthine oxidase (XO), an enzyme closely related to the pathophysiology of schizophrenia, was decreased in the occipital cortex of psychotic patients. To date, few studies have explored the relationship between the occipital lobe and schizophrenia with obsessive–compulsive symptoms based on neuroimaging; our findings provide a reference for future studies.

The brain region 58Postcentral_R, the postcentral gyrus, had a significantly lower MR-measured degree in SCH than in OCD (*p* = 0.018). The postcentral gyrus is an important brain region in the sensorimotor network, and neuroimaging studies have shown that obsessive–compulsive disorder is often associated with abnormal activity in this region (38). It has been observed that patients with OCD have reduced short-range positive functional connectivity (spFC) and long-range positive FC (lpFC) at rest and that the spFC of the left anterior central gyrus/posterior central gyrus can distinguish OCD patients from healthy controls (39). Le Jeune et al. (40) performed 18-fluorodeoxyglucose positron emission tomography (PET) in 10 patients with OCD and found hypermetabolism in the postcentral gyrus in non-stimulated OCD patients compared with healthy controls. Yu et al. (16) found that ALFF in the left postcentral gyrus was significantly lower in the OCD group than in the schizophrenia group or the healthy control group. There is no consensus on what kind of abnormal activity in the postcentral gyrus is associated with OCD. Wang et al. (41) found that gray matter volume (GMV) in the left postcentral gyrus was increased in individuals with high levels of schizophrenic and obsessive–compulsive traits compared to those with low scores for these traits.

The brain region 90Temporal_Inf_R, the inferior temporal gyrus, showed a significantly lower MR-measured degree in SCH than in OCD (*p* = 0.018). This brain region is known to play a crucial role in emotional and cognitive processing. Abnormalities in the structure and function of the temporal lobe can affect inhibitory control and executive function in patients with obsessive–compulsive disorder. Previous studies using structural magnetic resonance imaging have reported a decrease in the thickness of the temporal cortex in patients with obsessive–compulsive disorder (42). Additionally, Jinmin et al. (43) found that patients with obsessive–compulsive disorder exhibited a decrease in temporal lobe activation as measured by the blood oxygen level–dependent signal. A meta-analysis found an increase or decrease in resting-state brain activity in the temporal lobe, specifically the left superior temporal gyrus, in schizophrenia (44). Using resting-state functional magnetic resonance imaging, Liang et al. (45) found that the ALFF of the middle temporal gyrus was significantly reduced in childhood-onset schizophrenia with obsessive–compulsive symptoms (COSO) compared with schizophrenia without obsessive–compulsive symptoms (COS). Wang et al. (46) examined gray matter volume and cortical thickness in 22 schizophrenia patients with obsessive–compulsive symptoms and found that these patients had reduced cortical thickness in the right superior temporal gyrus compared with healthy controls. These results are consistent with the results of our study.

Clozapine is an important second-generation antipsychotic drug that has balanced antidopaminergic and anti-serotonergic properties and is used to treat schizophrenia. It is particularly useful for schizophrenia that has been resistant to other treatments. However, the most commonly observed adverse effect of clozapine treatment is OCS. Some studies have reported that as many as 35.9% of clozapine users subsequently develop OCS (47). In our study, we observed that the MR-measured degree of the 02Precentral_R brain region (precentral gyrus in the frontal lobe) was significantly reduced in the LDP group compared to the OCD group (*p* = 0.006). Compared with the CN group, the LDP group had a significantly reduced degree as measured by MR (*p* = 0.02). In the 47Lingual_L brain region (lingual gyrus in the occipital lobe), the degree was significantly higher in the LDP group (*p* = 0.002) and CN group (*p* = 0.0026) than in the OCD group. Similarly, in the 58Postcentral_R brain region (postcentral gyrus in the parietal lobe), the degree was significantly reduced in the LDP group compared to the OCD group (*p* = 0.009). Finally, in the 90Temporal_Inf_R brain region (inferior temporal gyrus in the temporal lobe), the degree was significantly reduced in the LDP group compared to the OCD group. These findings suggest that LDP treatment for schizophrenia may have beneficial effects on several brain regions, including the precentral gyrus, postcentral gyrus, lingual gyrus, and inferior temporal gyrus, which are all implicated in the pathological mechanism of OCD.

At present, the mechanism of clozapine inducing OCS is still unclear. Some studies have speculated that clozapine can induce OCS due to its strong innate antiserum effect, especially with 5-HT1C, 5-HT2A, and 5HT2C receptors (48). In addition to pharmacodynamic mechanisms, specific genetic characteristics may predispose schizophrenia patients to develop secondary OCS during treatment. One candidate polymorphism is in SLC1A1, the gene that encodes the neuronal glutamate transporter and is independently associated with genetic risk for OCD (49). Some scholars also propose that patients with OCS after clozapine treatment exhibit significant cognitive defects in areas such as visual recognition memory, impulse inhibition, perseverative responses, and set-switching ability and that these cognitive deficits are strongly associated with the severity of OCS (50). Therefore, cognitive defects may be related to the pathological mechanism of OCS occurrence. In terms of neurological influence, Şule Bıcakıay et al. studied 18 schizophrenia patients, including nine patients with clozapine-induced OCS [Clz OCS (+)], nine patients without OCS [Clz OCS (−)], and nine healthy controls. The study showed that WM integrity in several pathways, such as the cortico-striato-thalamo-cortical circuitry and orbitofrontal tracts, appears to be affected differently in patients with Clz-OCS (+). Different neuroplastic effects of clozapine leading to the occurrence of OCS in a subgroup of patients are possible and require further evaluation through longitudinal follow-up studies (51). However, there are still very few studies in this area.

However, this study still leaves some unanswered questions, such as what caused such different changes in connectivity in patients, and further investigation is needed. Due to the small number of patients with schizophrenia and obsessive–compulsive symptoms caused by the clinical use of olanzapine, only 12 patients were in the LDP group. In this study, the demographic characteristics of the LDP group were not significantly different from those of the other groups (*p* > 0.05); therefore, the LDP group was also included in this study and discussion. This may cause a certain deviation in the study results, and further studies with larger sample sizes are needed to improve the credibility of the study results.

## Conclusion

In conclusion, the aim of this study was to evaluate brain connectivity in patients with schizophrenia and obsessive–compulsive symptoms using diffusion tensor imaging (DTI). Significant differences in the degree of nodes as measured by MR were observed in the 25Frontal_Med_Orb_L, 47Lingual_L, 58Postcentral_R, and 90Temporal_Inf_R regions among the SCH, OCD, and CN groups (*p* < 0.05). In the LDP group compared to the OCD group, significant differences in brain regions 02Precentral_R, 47Lingual_L, 58Postcentral_R, and 90Temporal_Inf_R (*p* < 0.05) were observed. Our results indicate that the MR-measured degree of the 02Precentral_R, 25Frontal_Med_Orb_L, 47Lingual_L, 58Postcentral_R, and 90Temporal_Inf_R brain regions significantly differed among the CN, OCD, SCH, and LDP groups and that alterations in MR degree may be the underlying mechanism of obsessive–compulsive symptoms in schizophrenia, which provides some references for future studies on schizophrenia with obsessive–compulsive symptoms.

## Data availability statement

The raw data supporting the conclusions of this article will be made available by the authors, without undue reservation.

## Ethics statement

The studies involving human participants were reviewed and approved by Tongde Hospital of Zhejiang Province. The patients/participants provided their written informed consent to participate in this study. Written informed consent to participate in this study was provided by the participants’ legal guardian/next of kin.

## Author contributions

LLu conceived the study. LIi, HL, WL, and LG collected the data. YH and XX analyzed the data and drafted the paper. All authors contributed to the article and approved the submitted version.

## Funding

This research was supported by the National Natural Science Foundation of China (Grant No. 81403502), the Zhejiang Provincial Major Research Projects of Traditional Chinese Medicine (Grant No. 2018ZY002), and the Shanghai Traditional Chinese Medicine Science and Technology Plan (Grant No. 2022CX004).

## Conflict of interest

The authors declare that the research was conducted in the absence of any commercial or financial relationships that could be construed as a potential conflict of interest.

## Publisher’s note

All claims expressed in this article are solely those of the authors and do not necessarily represent those of their affiliated organizations, or those of the publisher, the editors and the reviewers. Any product that may be evaluated in this article, or claim that may be made by its manufacturer, is not guaranteed or endorsed by the publisher.
